# Filling the Antibody Pipeline in Allergy: PIPE Cloning of IgE, IgG_1_ and IgG_4_ against the Major Birch Pollen Allergen Bet v 1

**DOI:** 10.3390/ijms21165693

**Published:** 2020-08-08

**Authors:** Verena K. Köhler, Silvia Crescioli, Judit Fazekas-Singer, Heather J. Bax, Gerhard Hofer, Christina L. Pranger, Karin Hufnagl, Rodolfo Bianchini, Sabine Flicker, Walter Keller, Sophia N. Karagiannis, Erika Jensen-Jarolim

**Affiliations:** 1 Comparative Medicine, The Interuniversity Messerli Research Institute of the University of Veterinary Medicine Vienna, Medical University Vienna and University Vienna, Veterinärplatz 1, 1210 Vienna, Austria; verena.koehler@vetmeduni.ac.at (V.K.K.); judit.singer@ist.ac.at (J.F.-S.); christina.pranger@vetmeduni.ac.at (C.L.P.); karin.hufnagl@vetmeduni.ac.at (K.H.); Rodolfo.Bianchini@vetmeduni.ac.at (R.B.); 2 Institute of Pathophysiology and Allergy Research, Centre of Pathophysiology, Infectiology and Immunology, Medical University of Vienna, Währinger Gürtel 18–20, 1090 Vienna, Austria; sabine.flicker@meduniwien.ac.at; 3 St. John’s Institute of Dermatology, School of Basic & Medical Biosciences, King’s College London, 9th Floor, Tower Wing, Guy’s Hospital, London SE1 9RT, UK; silvia.crescioli@kcl.ac.uk (S.C.); heather.bax@kcl.ac.uk (H.J.B.); sophia.karagiannis@kcl.ac.uk (S.N.K.); 4 NIHR Biomedical Research Centre at Guy’s and St Thomas’s Hospitals and King’s College London, Guy’s Hospital, London SE1 9RT, UK; 5 School of Cancer & Pharmaceutical Sciences, King’s College London, 9th Floor, Tower Wing, Guy’s Hospital, London SE1 9RT, UK; 6 Institute of Molecular Biosciences, BioTechMed Graz, University of Graz, Humboldtstraße 50, 8010 Graz, Austria; gerhard.hofer@uni-graz.at (G.H.); walter.keller@uni-graz.at (W.K.); 7 Breast Cancer Now Research Unit, School of Cancer & Pharmaceutical Sciences, King’s College London, Guy’s Cancer Centre, London SE1 9RT, UK

**Keywords:** allergy, IgE, IgG_1_, IgG_4_, FcεRI, CD23, antibodies, blocking antibodies, in vitro, allergen immunotherapy

## Abstract

Birch pollen allergy is among the most prevalent pollen allergies in Northern and Central Europe. This IgE-mediated disease can be treated with allergen immunotherapy (AIT), which typically gives rise to IgG antibodies inducing tolerance. Although the main mechanisms of allergen immunotherapy (AIT) are known, questions regarding possible Fc-mediated effects of IgG antibodies remain unanswered. This can mainly be attributed to the unavailability of appropriate tools, i.e., well-characterised recombinant antibodies (rAbs). We hereby aimed at providing human rAbs of several classes for mechanistic studies and as possible candidates for passive immunotherapy. We engineered IgE, IgG_1_, and IgG_4_ sharing the same variable region against the major birch pollen allergen Bet v 1 using Polymerase Incomplete Primer Extension (PIPE) cloning. We tested IgE functionality and IgG blocking capabilities using appropriate model cell lines. In vitro studies showed IgE engagement with FcεRI and CD23 and Bet v 1-dependent degranulation. Overall, we hereby present fully functional, human IgE, IgG_1_, and IgG_4_ sharing the same variable region against Bet v 1 and showcase possible applications in first mechanistic studies. Furthermore, our IgG antibodies might be useful candidates for passive immunotherapy of birch pollen allergy.

## 1. Introduction

Birch pollen allergy is among the most common allergies in Central and Northern Europe, with a prevalence of up to 16% [1]. More than 90% of individuals allergic to birch pollen are sensitised to a single major allergen, Bet v 1 [1,2]. Therefore, Bet v 1 has been extensively studied for more than 30 years [3,4,5].

The molecular key player in any allergy is IgE, which interacts with IgE receptors FcεRI and CD23 on the surface of effector cells [6]. Binding to the high-affinity receptor FcεRI on mast cells and basophils is of major importance for the immediate allergic reaction: Upon allergen binding to IgE, FcεRs are cross-linked and cells release mediators that are responsible for typical allergy symptoms [7]. An allergic individual typically has a polyclonal IgE response, which allows receptor cross-linking by monomeric allergens. However, a cross-link of FcεRI loaded with monoclonal IgE is possible according to the concept of allergen-associated molecular patterns (AAMPs), e.g., by allergens with repetitive identical epitopes—such as tropomyosin—or the formation of aggregates of monovalent allergens [8,9]. Bet v 1 is known to non-covalently form dimers in a concentration-dependent manner [8,10]. Although protein aggregation is thought to increase immunogenicity [11], studies with Bet v 1 have shown both an increase and decrease of its allergenicity with multimer formation [10,12].

The low-affinity IgE receptor CD23 is mainly expressed on B cells, but its expression can also be found or stimulated on various other cell types, such as monocytes, macrophages, follicular dendritic cells, and epithelial cells [13,14]. In contrast to FcεRI, CD23 does not belong to the family of Ig receptors. It is a C-type lectin that binds several ligands besides IgE, such as CD21 and various integrins, and can directly interact with major histocompatibility complexes (MHC) [13,14,15]. CD23 has many functions depending on its ligand, form (soluble vs. membrane-bound), and subtype (CD23a vs. CD23b). With regards to allergy, two important functions have the capacity to regulate IgE synthesis in B cells and mediate allergen internalisation, processing, and presentation to T cells, also known as facilitated antigen presentation (FAP) [6].

Currently, the only effective treatment of birch pollen allergy—besides symptomatic relief medication—is active allergen immunotherapy (AIT) [16]. In this approach, increasing doses of allergen are administered to the patient and the immune response is shifted from allergy to tolerance. The development of tolerance is dependent on IgG_4_ antibodies to the allergen [17] and raised IgG_1_ and especially IgG_4_ antibody serum levels are considered to be useful biomarkers for the success of AIT [18,19]. Based on these observations, the first therapeutic IgG targeting an allergen for passive immunotherapy was recently developed [20].

Although advances have helped understand the mechanisms of AIT [18], questions regarding the full functional characteristics of IgGs remain unanswered. The capability of these antibodies to block allergen recognition by IgE is undisputed; however, their Fc-mediated functions are insufficiently explored [21]. Furthermore, current studies focus on IgG_4_ and its many unusual properties [22] rather than the IgG_1_ isotype, despite observed increases in the levels of both subclasses in individuals undergoing AIT. In addition, few mechanistic studies directly compare IgE, IgG_1_, and IgG_4_, likely due to limited availability of well-characterised recombinant antibodies produced in sufficient amounts.

Polymerase Incomplete Primer Extension (PIPE) cloning can be employed for rapid combination of any known variable antibody regions with the constant region sequences of any isotype to generate functional antibodies [23]. This has been applied in oncology to study antibodies of the most commonly used therapeutic agent isotypes IgG_1_ and IgG_4_ [24]. Furthermore, it has led to the recent emergence of IgE as a novel antibody isotype in cancer treatment in the AllergoOncology field [25,26].

We now applied PIPE cloning with the objective of deriving IgG_1_ and IgG_4_ isotypes from a known IgE antibody against the major birch pollen allergen Bet v 1 [27] and elucidating their target antigen engagement. Our main aim was to provide variable region-matched antibodies for mechanistic studies. These antibodies could help to understand the role of Bet v 1 aggregation on a cellular level and shed light on the Fc-mediated role of IgG antibodies in AIT. They furthermore might prove useful as candidates for passive immunotherapy.

## 2. Results

### 2.1. Biochemical Characterisation of PIPE-Cloned Anti-Bet v 1 Antibodies

**Figure 1:**
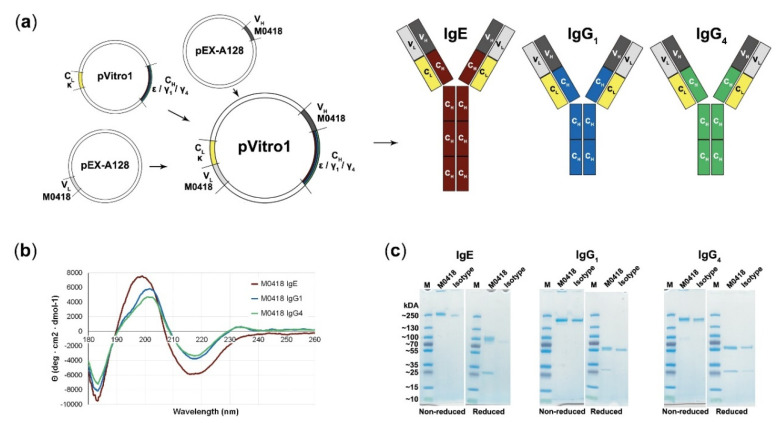
Polymerase Incomplete Primer Extension (PIPE) cloning of several antibody classes and confirmation of their integrity. (**a**) Schematic representation of the PIPE cloning method and cloned antibodies. Variable regions (M0418) are recombined with different constant regions to create constructs for IgE, IgG_1_, and IgG_4_, respectively. (**b**) Circular dichroism spectra of M0418 IgE, IgG_1_, and IgG_4_ confirmed correct antibody assembly. (**c**) SDS-PAGE analysing purity and size of antibodies, as compared to the respective isotype controls.

“M0418” anti-Bet v 1 variable heavy and light chain sequences [27] were successfully recombined with κ light chains and ε, γ_1_, or γ_4_ heavy chain sequences by PIPE cloning (see method schematic in Figure 1a) and expressed in the human Expi293F system.

CD spectroscopy was carried out in order to confirm antibody integrity (Figure 1b). Spectra showed a strong maximum at 199 (IgE)–201 (IgGs) nm and two minima at 183 and 218 nm (all isotypes). M0418 IgG antibodies furthermore showed a second slight maximum at 230 nm.

SDS-PAGE indicated high purity of our antibodies, showed migration patterns similar to a commercial isotype control and gave a first indication of the molecular weight of the antibodies, which was around 250 kDa for IgE and between 130–250 kDa for the IgGs (Figure 1c; see Figures S1–S4 for uncropped gel images).

SEC-MALS analysis confirmed this observation and yielded a molecular weight of 226 (IgE), 168 (IgG_1_), and 161 (IgG_4_) kDa (Table 1). Therefore, the measured molecular weight was higher than the expected theoretical molecular weight determined from the amino acid sequence.

### 2.2. Specificity Determination

**Figure 2:**
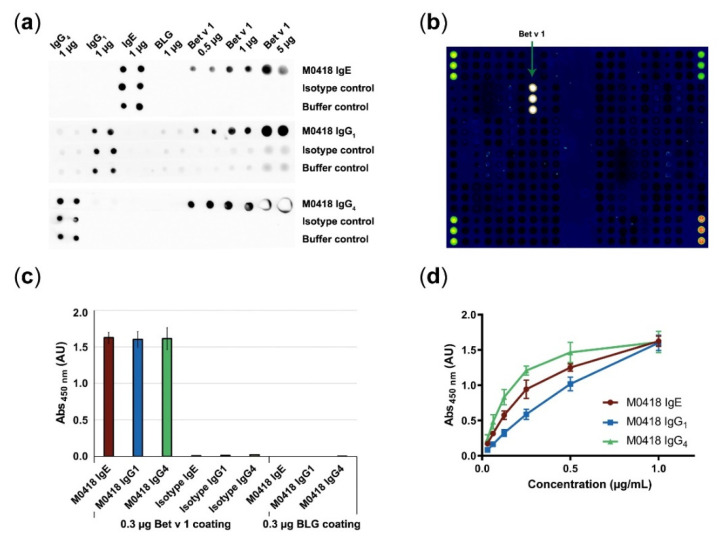
Confirmation of antibody specificity. (**a**) Specificity dot blot confirmed binding of M0418 antibodies to membrane-bound Bet v 1, but not to a control allergen (BLG); spotted antibodies act as an integrity control for the assay. (**b**) ImmunoCAP ISAC112 microarray confirmed binding of M0418 IgE to Bet v 1 but no other of the spotted 112 allergens. (**c**) ELISA confirmed specific binding of M0418 antibodies to Bet v 1, but not to the control allergen BLG; respective isotype controls were included as an assay integrity control. (**d**) ELISA showed concentration-dependent binding of M0418 antibodies to immobilised Bet v 1. Results in (**c**,**d**) are represented as means ± SD of three independent experiments.

We confirmed antibody specificity to immobilised Bet v 1 by dot blot, ImmunoCAP ISAC112 (Thermo Fisher Scientific, Waltham, MA, USA) microarray and in-house ELISA (Figure 2).

All M0418 isotypes recognised membrane-bound Bet v 1—but not the negative control allergen β-lactoglobulin (BLG)—in a dot blot (Figure 2a). The IgG_1_ control strips gave a faint background signal that can be attributed to unspecific binding of the secondary antibody. Therefore, another HRP-labelled antibody was used for IgG_1_ detection in all further experiments (i.e., ELISA).

M0418 IgE binding to Bet v 1 was furthermore confirmed in the ImmunoCAP ISAC112 microarray (Thermo Fisher Scientific, Waltham, MA, USA), where binding appeared to be exclusive to Bet v 1, i.e., it did not bind any of the other 111 allergens that were immobilised on the chip (Figure 2b).

Specificity was furthermore confirmed in an ELISA (Figure 2c), where M0418 antibodies bound Bet v 1, but not BLG. In addition, only M0418 antibodies—and not the respective isotype control antibodies—recognised Bet v 1.

Finally, we confirmed concentration-dependence of Bet v 1 binding in an ELISA for all M0418 isotypes (Figure 2d). Differences in the shape of the curves can be attributed to the different detection antibodies used.

### 2.3. M0418 IgG Inhibits Serum IgE Binding in a Quantitative ELISA

Next, we were interested in possible inhibitory functions of our IgG antibodies as well as utilising our IgE for Bet v 1—specific IgE quantification from human plasma.

**Figure 3:**
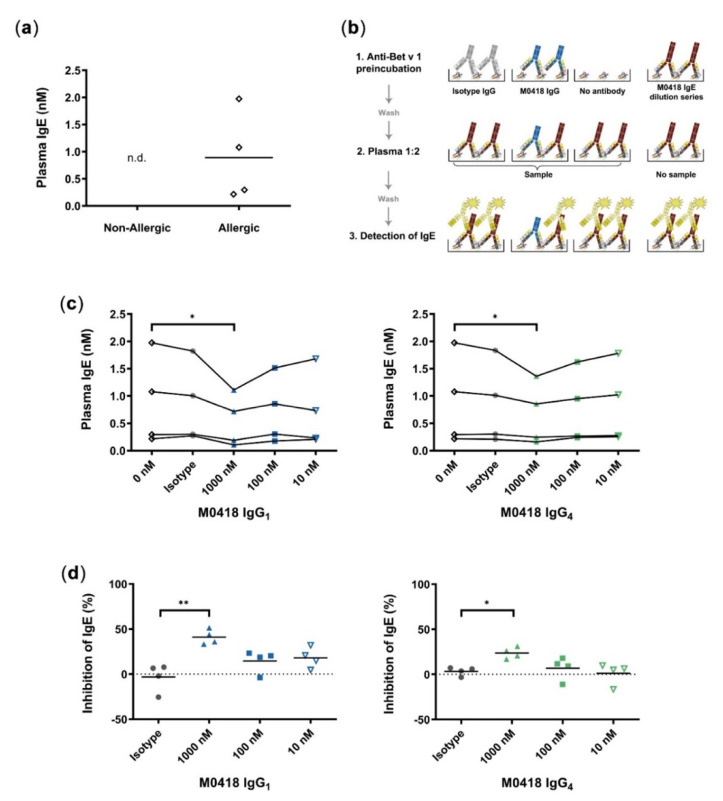
Quantitative inhibition ELISA. (**a**) Quantification of Bet v 1-specific IgE from plasma of allergic and non-allergic individuals; n.d., not detectable (below limit of detection). (**b**) Schematic of quantitative blocking ELISA. 1, M0418 IgG antibodies (blue), or isotype controls (grey) were added for binding to Bet v 1 coated on ELISA plates. 2, Plates were incubated with plasma from birch pollen allergic individuals and 3, bound IgE (red) was detected by HRP-labelled Anti-IgE antibodies (yellow). The IgE standard curve from a M0418 IgE dilution series allowed quantification of plasma IgE levels. This blocking ELISA scheme was used in (**c**) for quantification of plasma IgE levels of Bet v 1-allergic individuals. 1000 nM M0418 IgG antibodies significantly decreased the amount of IgE-binding in contrast to the respective isotype controls. (**d**) Normalisation of IgE inhibition per individual shows that the M0418 IgG antibodies significantly decreased binding of IgE to Bet v 1 by 20–50%. Each data point represents the mean value of one allergic individual, as determined in three separate experiments, hence *n* = 4. * *p* < 0.05, ** *p* < 0.01.

Therefore, we created a quantitative blocking ELISA, in which we used our recombinant M0418 IgE to set up a standard curve and determine the IgE plasma levels of four birch-pollen-allergic and non-allergic donors, respectively. The levels of IgE that were measured in the patients’ blood samples were between 0.2 and 2 nM, while those in serum samples from non-allergic donors were below the limit of detection in this assay (Figure 3a). In addition, we determined the capabilities of our IgG antibodies to block IgE binding to Bet v 1 in these allergic donor samples (see Figure 3b for a scheme describing the method). We chose relatively high maximum IgG concentrations to cover all possible IgE/IgG ratios that were reported in the literature [29].

M0418 IgG_1_ and IgG_4_—but not their respective non-specific isotype controls—could decrease the relative amount of plasma IgE binding to immobilised Bet v 1 at 1000 nM (Figure 3c). The normalisation of measurements per donor showed that antibodies significantly decreased binding of plasma IgEs to Bet v 1 by 20–50% (Figure 3d). M0418 IgG_1_ inhibited plasma IgE binding more effectively than M0418 IgG_4_.

### 2.4. M0418 IgE Upregulates FcεRI in Human LAD2 Mast Cells

We then investigated the Fc-mediated functionality of M0418 IgE in vitro by investigating interaction with the high-affinity IgE receptor FcεRI. Because IgE can upregulate FcεRI expression in mast cells [7,30], we studied FcεRI expression in the human mast cell line LAD2 upon M0418 IgE treatment [31].

**Figure 4:**
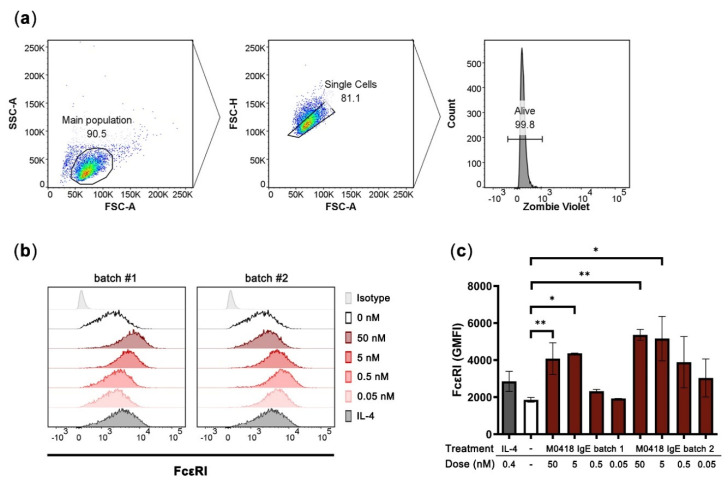
Upregulation of FcεRI in human LAD2 mast cells upon stimulation with M0418 IgE. (**a**) Gating strategy. (**b**) Representative histograms showing FcεRI expression of LAD2 cells. Two different batches of M0418 IgE were tested, IL-4 was included as a positive control. (**c**) FcεRI was upregulated in LAD2 cells after a 4-day-incubation with two production batches of recombinant M0418 IgE. SSC-A, side scatter area; FSC-A, forward scatter area; FSC-H, forward scatter height; Zombie Violet, viability dye; GMFI, geometric mean fluorescence intensity. Data are represented as means ± SD of at least three independent experiments. * *p* < 0.05, ** *p* < 0.01.

After four days of incubation with M0418 IgE, LAD2 cells showed a significant increase in FcεRI expression (Figure 4). The upregulation was dependent on the IgE concentration and more pronounced than upregulation triggered by IL-4 stimulation (positive control [32]). A maximum was reached at 5 nM of IgE. Furthermore, there was no significant difference between two batches of M0418 IgE with regard to receptor upregulation, indicating uniformity of our production system (Figure 4c).

### 2.5. Bet v 1 Internalisation is Mediated by M0418 IgE in U937 Monocytes

Next, we sought to study Fc-mediated functionality via the low-affinity IgE receptor CD23. We labelled Bet v 1 with the pH-sensitive dye pHrodo Green and studied allergen internalisation via IgE immune complex formation in: (a) IL-4-primed human monocytic U937 cells that upregulate cell surface CD23 [33], and (b) the CD23^hi^ human B-cell line RPMI-8866. Cells were either pre-treated with M0418 IgE or an isotype control to determine internalisation specificity. We furthermore added either Bet v 1 alone or Bet v 1 in combination with an anti-IgE antibody, to establish a positive control for Bet v 1 internalisation via artificial receptor crosslinking.

**Figure 5:**
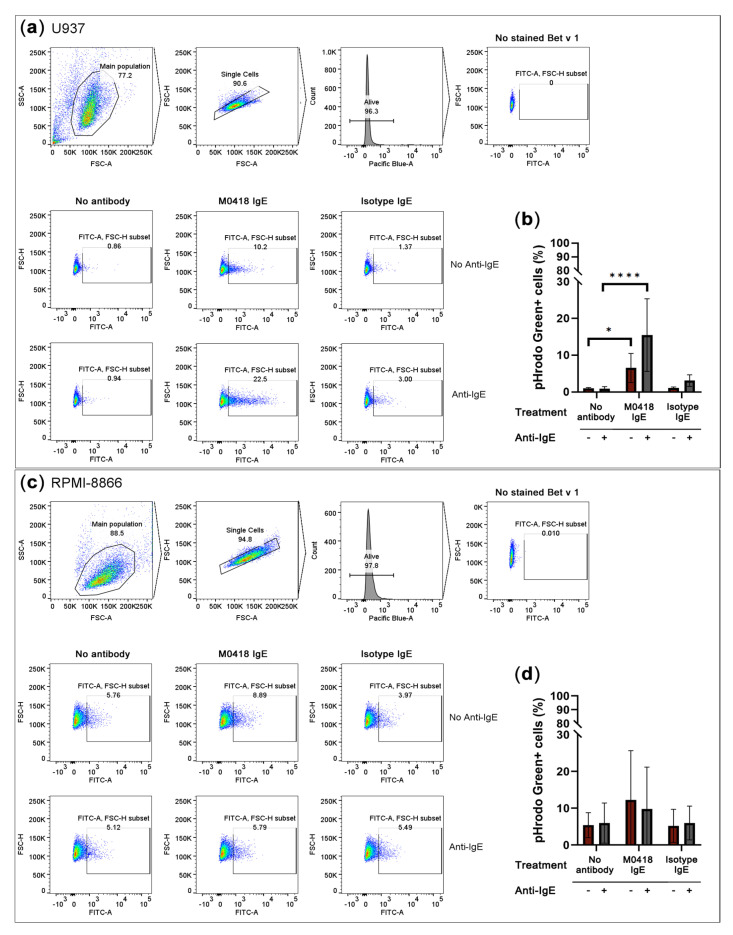
Overnight internalisation of pHrodo Green-labelled Bet v 1 by IL-4-primed U937 monocytes and the CD23^hi^ B-cell line RPMI-8866. (**a**) Gating strategy and representative flow cytometry data of U937 internalisation. (**b**) U937 monocytes internalised Bet v 1 in an IgE-dependent manner. (**c**) Gating strategy and representative flow cytometry data of RPMI-8866 internalisation. (**d**) Bet v 1-internalisation by RPMI-8866 cells was IgE-independent. SSC-A, side scatter area, FSC-A, forward scatter area, FSC-H, forward scatter height. Pacific Blue-A, area of Pacific Blue channel signal which indicates DAPI viability staining. Data are represented as means ± SD of at least three independent experiments. * *p* < 0.05, ** *p* < 0.01, *** *p* < 0.001, **** *p* < 0.0001.

Both of the cell lines expressed CD23, but not FcεRI, as confirmed by flow cytometry (Figure S5). In U937 monocytic cells, Bet v 1 internalisation was dependent on allergen-specific IgE recognition (Figure 5a,b). Artificial crosslinking via anti-IgE antibody further enhanced internalisation.

RPMI-8866 cells showed a high level of unspecific internalisation and, thus, internalised Bet v 1 independently of IgE (Figure 5c,d).

### 2.6. M0418 IgE Mediates Bet v 1-Degranulation in RBL-SX38 Basophils

Finally, we aimed to investigate the main effector function of our M0418 IgE, allergen-dependent degranulation. A well-established system for degranulation studies is the rat basophilic leukaemia cell line RBL-SX38, which is transfected with a fully functional human FcεRI [34]. We were curious as to whether we could trigger degranulation of cells that were sensitised with monoclonal antibody by addition of Bet v 1. To ensure the saturation of all Fcε Receptors on the cell surface, we used a sensitisation concentration of 5 nM IgE. This was furthermore in accordance with the values recommended in the literature [35].

**Figure 6:**
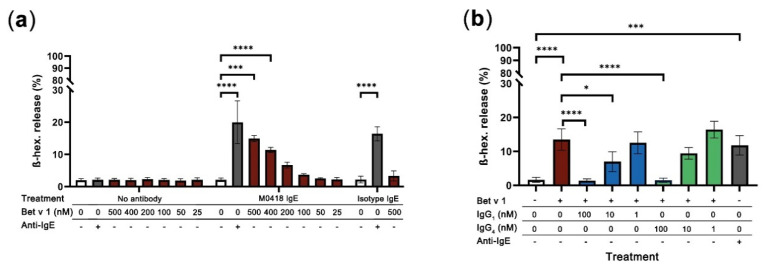
RBL-SX38 degranulation assay. (**a**) Bet v 1 caused degranulation of RBL-SX38 basophils sensitised with M0418 IgE—but not with isotype control IgE—in a concentration-dependent manner. Degree of degranulation was comparable with the artificial crosslinker positive control (Anti-IgE). (**b**) Inhibition of Bet v 1-dependent degranulation of RBL-SX38 cells pre-treated with M0418 IgE by M0418 IgG antibodies was dependent on blocking antibody concentration. Data are represented as means ± SD of at least three independent experiments. * *p* < 0.05, ** *p* < 0.01, *** *p* < 0.001, **** *p* < 0.0001.

Interestingly, Bet v 1 could crosslink FcεRI-bound allergen-specific IgE-but not non-specific isotype control IgE-and cause degranulation in RBL-SX38 cells (Figure 6a). Degranulation was concentration-dependent with regards to Bet v 1 and reached similar levels as those achieved with cross-linking RBL-SX38-bound IgE with polyclonal anti-IgE (positive control). Degranulation was significant with 400 and 500 nM of Bet v 1 and specific when compared to the isotype control.

When we tested whether our M0418 IgG antibodies could block epitopes on Bet v 1 and, thus, prevent degranulation of RBL-SX38 cells sensitised with M0418 IgE, both M0418 IgG_1_ and IgG_4_ blocked Bet v 1 degranulation in a concentration-dependent manner (Figure 6b). In both cases, 100 nM of IgG antibody were sufficient to completely inhibit degranulation caused by 400 nM Bet v 1.

## 3. Discussion

We successfully used PIPE cloning to produce fully human, recombinant anti-Bet v 1 IgE, IgG_1_, and IgG_4_ that share the same variable region. We confirmed antibody integrity and purity with CD spectroscopy, SDS-PAGE and SEC-MALS.

The shape of the presented CD spectra is typical for immunoglobulins—which consist mainly of β-sheets—and overall indicates correct assembly [36,37]. Deviations of the IgE spectrum from the IgG spectra can be attributed to the two additional constant domains in the Fc-region of IgE.

SDS-PAGE indicated a higher molecular weight than calculated from the sequence, which was confirmed by SEC-MALS. Such discrepancies between the theoretical and experimentally determined molecular weights may be attributed to antibody glycosylation. The fact that this effect was more pronounced for M0418 IgE than its IgG counterparts supports this notion, as IgE has substantially more glycosylation sites [38].

We confirmed specificity to Bet v 1 in a dot blot, allergen microarray and ELISA. ImmunoCAP ISAC112 data stringently confirmed the original observation that the M0418 variable region has very limited cross-reactivity with other structurally related PR-10 allergens [27].

After thorough characterisation, we approached functional studies and utilised all three M0418 antibody isotypes in a quantitative inhibition ELISA using allergic patient donor plasma. The Bet v 1-specific plasma IgE levels detected from allergic donors were consistent with those reported in the literature [29,39,40]. The partial blocking of Bet v 1 recognition by polyclonal patient-derived antibodies is likely due to the limited epitope coverage of our monoclonal antibodies and thus to incomplete blockade of multiple epitopes on Bet v 1 recognised by patient IgEs [41]. However, our overall results were in accordance with Bet v 1-inhibition ELISAs reported by others [42,43], see a comparison of relevant assay parameters in Appendix A in Table A1. Of note, M0418 IgG_4_ showed a weaker blocking capacity than its IgG_1_ counterpart, which can possibly be attributed to its unique property of Fab arm exchange with other IgG antibodies [22].

Furthermore, M0418 IgE was fully functional with regards to Fc-mediated effects in appropriate FcεRI and CD23 in vitro models.

It is well known that IgE levels positively correlate with the FcεRI expression on mast cells. Accordingly, in our model, culturing human LAD2 mast cells with M0418 IgE for several days resulted in an increase of of FcεRI on the cell surface, which are likely attributed to receptor upregulation [30]. Only few similar studies exist, mainly due to the lack of the necessary tools and we thus propose that M0418 IgE could be used as a practical reagent to improve mediator release of mast cells and other effector cells in in vitro. In fact, M0418 IgE incubation reached the plateau of receptor upregulation at similar concentrations as in previous reports [30].

The findings of our internalisation studies were rather surprising: Bet v 1 internalisation appeared to be strictly antibody-dependent in the case of U937 monocytes, and unspecific and antibody-independent in the case of RPMI-8866 B cells. Based on the nature of these cell types and previous findings in B cells showing their IgE-dependent allergen internalisation and presentation to T cells, we would have assumed opposite results [44,45,46]. To our knowledge, only one study exists, which investigates Bet v 1 internalisation by monocytes and B cells [47]. However, the analysis of these cell types is very brief, as the focus of the work lay on basophils. Furthermore, a comparison of our results with these findings is very difficult, as the authors analysed cellular subsets from PBMCs of allergic and non-allergic donors rather than cell lines treated with recombinant antibodies. In their findings, B cells of both allergic and non-allergic donors to a small, but similar extent, internalised Bet v 1. Monocytes, however, showed an overall strong and unspecific internalisation of Bet v 1, which is even more pronounced in an allergic setting. It is clear from the lack of available data, that these observations require additional studies. With our antibodies it should now be possible to investigate the antibody-dependence of Bet v 1 internalisation in detail. We suggest additional studies with several model cell lines as well as primary cells and investigating not only the role of IgE, but also IgG antibodies in internalisation or inhibition thereof.

In addition to these CD23-mediated internalisation studies, we were able to show allergen-dependent, IgE-mediated degranulation of RBL-SX38 basophils. M0418 IgE-mediated degranulation was caused by binding of increasing concentrations of Bet v 1. This finding appears to go against the textbook knowledge that a single-epitope allergen can only be crosslinked by polyclonal IgE. However, the formation of allergen multimers would not only theoretically allow crosslinking of monoclonal antibodies [8], but was recently shown in detail in a study with Timothy grass pollen allergen [48]. Recent degranulation studies with an engineered Bet v 1 trimer confirm this notion [44]. Additional studies of our group with monoclonal antibodies against the major cow’s milk allergen BLG confirm these findings [49]. It is furthermore well-known that Bet v 1 is prone to form dimers, as was shown previously [11,50].

Overall, we present, for the first time, a systematic characterisation of IgE, IgG_1_, and IgG_4_ sharing the same variable region targeting the major birch pollen allergen Bet v 1. Besides proving IgG blocking capabilities of our antibodies, we also confirmed full functionality of M0418 IgE in vitro.

Our findings may have raised further questions with regard to Bet v 1 aggregation and internalisation by B cells and monocytes but we now for the first time present the appropriate tools to address such mechanistic questions in vitro.

## 4. Materials and Methods

### 4.1. Cell Line Cultivation and Maintenance

Expi293F cells (cat# A14527, Thermo Fisher Scientific, Waltham, MA, USA) were cultivated in 30 or 125 mL of Expi293 Expression Medium (cat# A1435101, Gibco, Thermo Fisher Scientific, Waltham, MA, USA) in 125 mL or 500 mL baffled Erlenmeyer flasks with vented caps (Corning, New York, NY, USA), respectively, at 37 °C, 8% CO_2_, according to the manufacturer’s instructions. LAD2 [31] (kind gift of A. Kirshenbaum and D. Metcalfe, National Institute of Allergy and Infectious Diseases, Bethesda, MD, USA) were grown in StemPro-34 medium (cat# 61870044, Gibco, Thermo Fisher Scientific, Waltham, MA, USA) that was supplemented with 13 mL StemPro-34 Nutrient Supplement (cat# 10639011, Gibco, Thermo Fisher Scientific, Waltham, MA, USA) per 500 mL medium, L-Glutamine (2 mM), penicillin (100 U/mL)/streptomycin (100 µg/mL), and rhSCF (100 ng/mL; cat#11343327, Immunotools GmbH, Friesoythe, Germany) in T75 cell culture flasks (Corning, New York, NY, USA) at 37 °C, 5% CO_2_. RBL-SX38 [34], RPMI-8866 (ECACC 95041316), and U937 cells (ATCC^®^ CRL-1593.2™) were cultivated in RPMI 1640 medium with GlutaMAX^TM^ (cat# 618700, Thermo Fisher Scientific, Waltham, MA, USA), supplemented with 10% FBS (Gibco, Thermo Fisher Scientific, Waltham, MA, USA), and maintained in T75 flasks (Corning, New York, NY, USA) at 37 °C, 5% CO_2_.

### 4.2. Allergens

Recombinant Bet v 1 (Bet v 1.0101) was produced in *E. coli* and quality was tested, as previously described [51,52]. Commercially available milk allergen β-lactoglobulin (BLG; cat# L01030, Sigma-Aldrich, St. Louis, MO, USA) was used as a negative control allergen in some of the experiments.

### 4.3. pHrodo Green-Labelling of Bet v 1

Bet v 1 was labelled with a pHrodoTM Green STP ester kit (cat# P35369, Life Technologies, Carlsbad, CA, USA) according to the manufacturer’s instruction. Briefly, 200 µg of Bet v 1 was labelled at a concentration of 1 mg/mL in 0.1 M bicarbonate buffer. For the labelling reaction, a molar dye: protein ratio of approximately 10:1 was used. The reaction mixture was incubated at room temperature for 1 h, protected from light. Tube was inverted every 15 min. to ensure mixing. After labelling, pHrodo Green-Bet v 1 was dialysed against phosphate-buffered saline (PBS) in order to remove excess dye using a Slide-A-Lyzer™ MINI Dialysis Device with a cut-off of 3.5 kDa (cat# 88401, Thermo Fisher Scientific, Waltham, MA, USA), overnight at 4 °C. Buffer was exchanged and the sample was dialysed for another 4.5 h at 4 °C. Labelled Bet v 1 was stored at 4 °C until used.

### 4.4. PIPE Cloning

M0418 variable region sequences were obtained from a previously published human IgE sequence [27]. If necessary, amino acid sequences were translated into nucleotide sequences and manually optimised for expression in human cells using various online tools included in the Sequence Manipulation Suite [53]. Optimised sequences were synthesised commercially and provided in pEX_A128 vectors (Eurofins Genomics AT GmbH, Vienna, Austria). PIPE cloning was carried out as previously described [23,54]. Briefly, light chain regions (M0418L, κ) and heavy chain regions (M0418H, ε/γ1/γ4) were amplified in four separate PIPE PCRs using Phusion Flash High-Fidelity PCR master mix (cat# F548S, Thermo Fisher Scientific, Waltham, MA, USA) and a MyCyclerTM thermal cycler (Bio-Rad Laboratories, Hercules, CA, USA). The respective template/primer combinations as well as PCR extension times are listed in Table 2. After confirmation of the expected size via agarose gel electrophoresis, the PCR products were digested for 1–2 h with DpnI (cat# R0176S, New England Biolabs, Ipswich, MA, USA) in the provided CutSmart^®^ buffer at 37 °C, followed by 20 min. enzyme deactivation at 80 °C. Digested PCR products were then mixed in a 1:1:1:1 ratio and incubated at room temperature for at least 2 h up to overnight for ligation. NEB 10-beta Competent *E. coli* (cat# C3019, New England Biolabs, Ipswich, MA, USA) was transformed with the resulting pVitro1-hygro-mcs constructs according to the manufacturer’s instructions. Selection was carried out on LB-agar plates supplemented with 150 µg/mL hygromycin B (cat# 1287, Carl Roth GmbH & Co KG, Karlsruhe, Germany). Colonies were screened for constructs via colony PCR. The integrity of construct antibody sequences was finally confirmed by Cycle sequencing (Mix2seq overnight kit; Eurofins Genomics AT GmbH, Vienna, Austria). The resulting antibody sequences are summarised in Appendix A in Table A2 and heavy and light chain sequences are deposited at GenBank (accession numbers MT435130, MT435131, MT435132 and MT435133).

### 4.5. Expression of Constructs and Antibody Purification

Expi293F cells were transfected with 30 µg of plasmid DNA according to the manufacturer’s instructions, with some modifications: 50 µg/mL of hygromycin B (cat# 1287, Carl Roth GmbH & Co KG, Karlsruhe, Germany) were added 72 h after transfection to further enhance production. Supernatant was harvested on day 7 after transfection by two consecutive centrifugation steps at low speed (280 x g, 7 min.), followed by a final centrifugation step at 3500 x g for 35 min. Supernatant was then filtered through a 0.22 µm PES membrane filter (cat# 124-0020, Thermo Fisher Scientific, Waltham, MA, USA) and diluted 1:2 with PBS prior to purification. The antibodies were purified via affinity chromatography using either a HiTrap KappaSelect column (IgE purification, 1 mL; GE Healthcare Life Sciences, Marlborough, MA, USA) or a HiTrap Protein A HP antibody purification column (IgG_1_ and IgG_4_ purification, 1 mL; GE Life Sciences) and an AEKTA chromatography system (GE Life Sciences/Amersham Biosciences, Marlborough, MA, USA). The antibodies were eluted from the column with 0.1 M glycine, pH 2.5, step elution (IgE) or 0.1 M citric acid, pH 3, gradient elution (IgG antibodies), respectively. Eluate was collected in 1 mL fractions and pH was immediately neutralised with 100 µL of Tris-HCl, pH 9.3. Antibody-Containing fractions were pooled and dialysed overnight at 4 °C against PBS using Tube-O-Dialyzer medi dialysis devices with a molecular cut-off of 15 kDa (cat# 786-618, GBiosciences, St. Louis, MO, USA). Concentration was then determined by measuring the UV absorption at 280 nm with a UV/VIS spectrophotometer (DeNovix DS-11 FX+, DeNovix Inc., Wilmington, DE, USA), using the respective extinction coefficients, as determined from the protein sequence. Antibody solutions were sterilised by passing them through a 0.2 µm PES filter (cat# 16532, Sartorius AG, Göttingen, Germany) and stored at 4 °C until use.

### 4.6. Determination of Antibody Integrity

#### 4.6.1. Circular Dichroism Spectroscopy

The circular dichroism (CD) spectra of antibodies were measured in 75 mM phosphate buffer (pH 7.2) with a 0.1 mm quartz cuvette with on a Jasco J-1500 spectropolarimeter (Jasco International Co., Tokyo, Japan) at room temperature. Sample concentrations were between 0.2 and 0.6 mg/mL. Spectra were obtained from 260–180 nm, data pitch 0.2 nm, at a scan speed of 20 nm/min., with a response (D. I. T., digital integration time) of 8s. Data represent the average of 5 accumulated measurements and they are given as mean residue ellipticity.

#### 4.6.2. SDS-PAGE

2–3µg of M0418 IgE, IgG_1_ and IgG_4_ or the respective commercially available isotype controls (human plasma IgE, human myeloma IgG_1_, and human myeloma IgG_4_, cat# 16-16-090705, 16-16-090707-1M, and 16-16-090707-4M, respectively, Athens Research & Technology, Athens, GA, USA) were mixed with Laemmli loading buffer with and without β-mercaptoethanol (reducing vs. non-reducing conditions), respectively, and denatured at 95 °C for 5 min. Samples were loaded into 50 µL pockets of a 4–15% gradient polyacrylamide gel (Mini-PROTEAN^®^ TGX™ precast gel, cat# 4561084, Bio-Rad Laboratories, Hercules, CA, USA) and gel was run in SDS-Tris-glycine buffer at 200 V for 30–40 min. For size determination, PageRuler Plus prestained protein ladder (cat# 26619, Thermo Fisher Scientific, Waltham, MA, USA) was run on each gel. After electrophoresis, gel was stained with SimplyBlue safe stain (cat# LC6060, Thermo Fisher Scientific, Waltham, MA, USA) according to the manufacturer’s instructions.

#### 4.6.3. SEC-MALS

The samples in PBS were injected onto a SEC column (Superdex 200 Increase 10/300, GE Healthcare Life Sciences, Marlborough, MA, USA) at a flow rate of 0.5 mL/min. at 7 °C, detected at 280 nm, and coupled to a multi angle light scattering detector (miniDAWN TREOS, Wyatt, Santa Barbara, CA, USA).

### 4.7. Specificity Testing

#### 4.7.1. Dot Blot

Bet v 1 (0.5, 1, and 5 µg), BLG (1 µg), and isotype antibodies (1 µg; human plasma IgE, human myeloma IgG_1_ and human myeloma IgG_4_, cat# 16-16-090705, 16-16-090707-1M, and 16-16-090707-4M, respectively, Athens Research & Technology, Athens, GA, USA) were spotted onto a nitrocellulose membrane (0.2 µm nitrocellulose, cat# 10600001, GE Healthcare Life Sciences, Marlborough, MA, USA) in duplicate and dried overnight. Membrane was cut into strips and blocked with wash buffer (PBS with 0.1% Tween 20) supplemented with 5% skimmed milk powder (Heirler Genovis GmbH, Radolfzell am Bodensee) for 1 h. The membrane was washed with wash buffer three times and incubated either with antibody diluted with wash buffer to a final concentration of 1 µg/mL (i.e., M0418 IgE, IgG_1_, IgG_4_, or the respective isotype control) or wash buffer only for 1 h. Strips were briefly rinsed with wash buffer, three times, and then washed for 5 min. The strips were then incubated with the respective HRP-labelled detection antibodies diluted with wash buffer (anti-hu-IgE, 1:6000, cat# A18793, Invitrogen; anti-hu-IgG_1_, 1:6000, cat# 9054-05, Southern Biotech, Birmingham, AL, USA; anti-hu-IgG_4_, 1:10 000, cat# 9200-05, Southern Biotech, Birmingham, AL, USA, respectively) for another 1 h. Strips were again briefly rinsed, three times and then washed for another 10 min. For signal detection, Clarity Max Western ECL substrate (cat# 1705062, Bio-Rad Laboratories, Hercules, CA, USA) was prepared and then added to the membrane according to the manufacturer’s instructions. Membrane pictures were acquired using a ChemiDoc^TM^ Touch Imaging System (Bio-Rad Laboratories, Hercules, CA, USA). All incubation and wash steps were carried out at room temperature.

#### 4.7.2. ImmunoCAP ISAC112

In addition to dot blot and ELISA, M0418 IgE specificity was tested using the ImmunoCAP ISAC112 microarray (Thermo Fisher Scientific, Waltham, MA, USA), strictly following the manufacturer’s instructions. A total of 1 µg M0418 IgE was used in the test, diluted with non-allergic serum.

#### 4.7.3. ELISA

Maxisorp 96-well plates (Thermo Fisher Scientific, Waltham, MA, USA) were coated with 0.3 µg of Bet v 1 or BLG (100 µL/well; concentration of 3 µg/mL) in bicarbonate buffer, pH 9.6, overnight at 4 °C. Liquid was removed and the plate washed with wash buffer (Tris-Buffered saline, TBS with 0.05% Tween 20). Plate was then blocked with 200 µL/well wash buffer with 1% BSA for 2 h. Plate was then washed twice and sample or control antibodies (either M0418 IgE, IgG_1_, or IgG_4_ and the respective myeloma isotype controls, see Section 4.7.1 above) diluted in wash buffer were added: Either at a final concentration of 1 µg/mL (100 µL per well; added to Bet v 1 and BLG-coated plate wells) or in a 1:2 dilution series starting with 1 µg/mL to 0.03 µg/mL (M0418 antibodies only; Bet v 1-coated wells only). After 1 h of incubation, plate was washed with wash buffer three times and incubated with the respective HRP-labelled detection antibodies (anti-hu-IgE, 1:6000, cat# A18793, Invitrogen, Carlsbad, CA, USA; anti-hu-IgG_1_, 1:1000, cat# A10648, Invitrogen, Carlsbad, CA, USA; anti-hu-IgG_4_, 1:24,000, cat# 9200-05, Southern Biotech, Birmingham, AL, USA, respectively) for another 1 h. The plate was again washed three times and 100 µL TMB/well (cat# 00-4201-56, Invitrogen, Carlsbad, CA, USA) was added. The reaction was stopped with 100 µL/well 1 M H_2_SO_4_ and absorbance at 450 nm as well as 630 nm (reference wavelength) was measured using an Infinite 200M PRO plate reader (Tecan, Männerdorf, Switzerland). All of the incubation and wash steps were carried out at room temperature, unless stated otherwise. Each condition was tested in triplicate and each experiment was repeated at least three times. The values corrected by the reference wavelength were used for analysis. The limit of detection (LOD) was defined as the mean of the blank plus three times the corresponding standard deviation. Raw values below the LOD were excluded from the analysis. Measurements from each well were blank-corrected and the mean values of replicate wells of each experiment were calculated with Excel 365 (Microsoft Corporation, Albuquerque, NM, USA).

### 4.8. Quantitative Plasma IgE Blocking ELISA

Maxisorp 96-well plates (Thermo Fisher Scientific, Waltham, MA, USA) were coated with 0.17 µg Bet v 1/well in coating buffer (bicarbonate buffer, pH 9.6) overnight at 4 °C. The plates were washed with wash buffer (TBS with 0.05% Tween 20) once and then blocked with wash buffer supplemented with 1% BSA for 2 h at room temperature. Sample wells (as opposed to standard row wells, which were blocked further) were washed with wash buffer four times and incubated with 10, 100, or 1000 nM of M0418 IgG_1_ or IgG_4_, 1000 nM of the respective isotype control (human myeloma IgG_1_ and IgG_4_, cat# 16-16-090707-1M and 16-16-090707-4M, respectively, Athens Research & Technology, Athens, GA, USA; stock concentration was determined via UV/VIS spectroscopy prior to preparing the working solution) or wash buffer, respectively, for 1 h. The plate was washed four times and 100 µL of 1:2 diluted plasma samples from birch pollen allergic individuals was added to sample wells. A 1:2 dilution series of M0418 IgE was prepared in the standard row wells, ranging from 0.5 nM to 0.007 nM and 3 nM to 0.05 nM, respectively; matching the expected IgE levels in the plasma samples). After 1.5 h of incubation, the plate was washed four times and HRP-labelled detection antibody added (anti-hu-IgE, 1:6000, cat# A18793, Invitrogen, Carlsbad, CA, USA). After another 1 h of incubation wash step was repeated and 100 µL TMB/well (cat# 00-4201-56, Invitrogen, Carlsbad, CA, USA) were added. Reaction was stopped with 100 µL of 1 M H_2_SO_4_ and absorbance at 450 nm and 630 nm (reference wavelength) was measured using an Infinite 200M PRO plate reader (Tecan, Männerdorf, Switzerland). IgE in plasma of four non-allergic donors was quantified in the same manner, but without the addition of any blocking antibodies. All of the incubation and wash steps were carried out at room temperature, unless stated otherwise. Each condition was tested in duplicate and each experiment was repeated at least three times. Reference-Wavelength corrected values were used for analysis. Values below the LOD were excluded from the analysis. Measurements from each well were corrected by the blank and duplicate means were calculated with Excel 365 (Microsoft Corporation, Albuquerque, NM, USA). Further statistical analysis was carried out with GraphPad Prism version 8 (GraphPad Software LLC, San Diego, CA, USA).

### 4.9. In Vitro Experiments

#### 4.9.1. FcεRI Upregulation of LAD2 Cells

A total of 1.5 x 10^5^ LAD2 cells (500 µL of 3 x 10^5^ cells/mL suspension) was incubated with 0.5, 5, or 50 nM of M0418 IgE, 6 ng/mL of IL-4 (positive control) or medium, respectively, in a 24-well plate. The cells were harvested after four days of incubation at 37 °C, 5% CO_2_ and washed with PBS twice. Cells were then stained with Zombie Violet (1:500 final; cat# 423114, Biolegend, San Diego, CA, USA) for 15 min., followed by co-incubation with APC-Cy7-labelled anti-FcεRI antibody (cat# 334632, clone# AER-37 (CRA-1), Biolegend, San Diego, CA, USA) or the respective isotype control antibody (IgG2b, kappa, mouse; cat# 400328, clone# MPC-11, Biolegend, San Diego, CA, USA) for another 15 min. The cells were then washed with Hank’s Balanced Salt Solution (HBSS; Gibco, Thermo Fisher Scientific, Waltham, MA, USA), centrifuged at 300 x g for 5 min., and then re-suspended in 200 µL of HBSS for flow cytometric analysis. 10,000 events were acquired using a FACSCanto II (BD Biosciences, Franklin Lakes, NJ, USA) and further analysis was carried out in FlowJo^TM^ version 10 (FlowJo LCC, Ashland, OR, USA) and Graphpad Prism version 8 (GraphPad Software LLC, San Diego, CA, USA).

#### 4.9.2. Internalisation of Labelled Bet v 1

U937 cells were primed with 10 ng/mL IL-4 for 48 h, twice, to upregulate CD23, as previously described [55]. Expression of CD23 and FcεRI was determined via flow cytometry while using the same staining protocol as in Section 4.9.1 but the following antibody panel: APC-Cy7-labelled anti-FcεRI antibody (cat# 334632, clone# AER-37 (CRA-1), Biolegend, San Diego, CA, USA), APC-labelled anti-CD23 antibody(cat# 338513, clone# EBVCS-5, Biolegend, San Diego, CA, USA), isotype control antibodies: APC-Cy7-labelled IgG2b, kappa, mouse (cat# 400328, clone# MPC-11, Biolegend, San Diego, CA, USA), and APC labelled IgG1, kappa, mouse (cat# 17-4714-42, clone#P3.6.2.8.1, eBioscience). Compensation was carried out with compensation beads (UltraComp eBeads^TM^, cat# 01-2222-42, Thermo Fisher Scientific, Waltham, MA, USA). LAD2 cells were included as a FcεRI-positive, CD23-negative staining control. For the determination of Bet v 1 internalisation, U937 cells and RPMI-8866 cells were harvested and washed with assay buffer (HBSS supplemented with 1% FBS) once. 100 µL containing 1 x 10^5^ cells were seeded into FACS tubes and 5 µg of M0418 IgE or isotype control IgE (NIP IgE, cloned in pVitro1, as previously described [23]) was added. One set of cells was left untreated. After 30 min. of incubation at 37 °C, cells were washed with assay buffer and 500 nM of labelled Bet v 1, diluted in assay buffer was added with or without additional crosslinker (polyclonal rabbit anti-IgE at a final concentration of 1.5 µg/sample; cat# A0094, Dako, Glostrup, Denmark). The cells were incubated with allergen for 1 h on ice, in the dark. Cells were again washed with assay buffer, re-suspended in 300 µL of medium supplemented with 2% FBS and incubated overnight at 37 °C, 5% CO_2_. After 16–18 h of incubation, the cells were washed and re-suspended in 200 µL of assay buffer with DAPI (final concentration: 3 µM; cat# 422801, Biolegend, San Diego, CA, USA) and subjected to flow cytometric analysis. 10,000 events were acquired using a FACSCanto II (BD Biosciences, Franklin Lakes, NJ, USA) and further analysis was carried out in FlowJo^TM^ version 10 (FlowJo LLC, Ashland, OR, USA) and GraphPad Prism version 8 (GraphPad Software LLC, San Diego, CA, USA).

#### 4.9.3. RBL-SX38 Degranulation Assay and Blocking of Degranulation

To determine cell degranulation, β-hexosaminidase release of RBL-SX38 cells was measured, as described previously [25]. Briefly, 1 × 10^4^ RBL-SX38 cells (in 100 µL medium) were seeded into the wells of a flat-bottom 96 well cell culture plate (Thermo Fisher Scientific, Waltham, MA, USA, Waltham, MA, USA) and incubated overnight to attach to the plate. The cells were briefly washed with assay buffer (HBSS supplemented with 1% of FBS) and sensitised with 5 nM of M0418 IgE or isotype control IgE (NIP IgE, cloned in pVitro1 as previously described [23]) for 2–3 h. Cells were washed three times with 200 µL assay buffer per well and stimulated with 5–500 nM of Bet v 1, 1.5 µg/mL polyclonal rabbit Anti-IgE (positive control; cat# A0094, Dako, Glostrup, Denmark) or buffer only (negative control) in a volume of 100 µL. Furthermore, a Triton-X control was included as a reference (100% degranulation). Cells were stimulated for 45 min. and assay plate was then centrifuged at 400 x g for 5 min. at 4 °C. 25 µL supernatant were transferred to a black 96 well plate (FluoroNunc^TM^, cat# 437796, Thermo Fisher Scientific, Waltham, MA, USA, Waltham, MA, USA). The samples were diluted with 25 μL of assay buffer and 50 µL of fluorogenic substrate (1 mM 4-methylumbelliferyl N-acetyl-b-D-glucosaminide in 100 mM citrate buffer, pH 4.5, with 0.1% DMSO) were added. The plate was incubated at 37 °C for 1 h in the dark and the reaction quenched with 100 μL of 0.5 M Trizma^®^ base (cat#6066, Sigma-Aldrich, St. Louis, MO, USA), pH 8.2, per well and fluorescence signal was measured using a FLUOstar Omega Microplate Reader (BMG Labtech, Ortenberg, Germany); excitation at 350 nM, emission at 450 nm. In the case of blocking experiments, stimulation was carried out with 400 nM of Bet v 1 with or without 1, 10, or 100 nM of M0418 IgG_1_ or IgG_4_, respectively. All of the measurements were made in quadruplicates and experiments were repeated at least three times. The signal gain was adjusted to 90% for the well giving the highest signal (Triton-X-lysed cells). The limit of detection (LOD) was defined as the mean of the respective blank (regular buffer vs. Triton-X) plus three times the corresponding standard deviation. Raw values that were below the limit of detection were eliminated from the analysis. All other values were then blank-corrected, normalised to the 100% degranulation control (Triton-X lysed cells) and their mean values were used for further statistical analysis. All of the incubation steps were carried out at 37 °C, 5% CO_2_, if not indicated otherwise. Basic calculations were carried out in Excel 365 (Microsoft Corporation, Albuquerque, NM, USA) and further statistical analysis was done in GraphPad Prism version 8 (GraphPad Software LLC, San Diego, CA, USA).

### 4.10. Patient Samples

Plasma samples from birch pollen-allergic individuals were obtained with their informed consent. Experiments with blood samples were in accordance with the Helsinki declaration of 1975 and they were approved by the institutional ethics committee of the Medical University of Vienna (EK number 2007/2016).

### 4.11. Statistical Analysis

For outlier identification, ROUT analysis (*Q* = 1%) was carried out. Data were then analysed via repeated measures one-way or two-way ANOVA or a corresponding mixed-effects model, as appropriate, followed by Dunnett’s multiple comparisons test. All of the statistical analyses were carried out using GraphPad Prism version 8 (GraphPad Software LLC, San Diego, CA, USA).

## 5. Conclusions

Overall, we present, for the first time, the rapid PIPE cloning of fully human, variable region-matched recombinant IgE, IgG_1_, and IgG_4_ antibodies specific to Bet v 1. Our IgE engaged with high and low affinity FcεRs and stimulated FcεRI upregulation in culture, while our IgG antibodies effectively inhibited IgE-mediated degranulation in vitro. Therefore, these basic tools may help to systematically investigate Fc-mediated effects of IgE versus IgG antibodies in allergy or tolerance at a cellular level. Future studies may prove our IgG_1_ and IgG_4_ to be useful candidates for passive immunotherapy of birch pollen allergy.

## Figures and Tables

**Table 1:** Biochemical characteristics of recombinant M0418 antibodies.

Sample	Extinction Coefficient ^a^ (M^−1^, cm^−1^)	Theoretical Molecular Weight, Unglycosylated ^a^ (kDa)	Measured Molecular Weight ^b^ (kDa)
M0418 IgE	294,940	180	226 (±0.7%)
M0418 IgG_1_	251,360	150	168 (±0.7%)
M0418 IgG_4_	251,360	150	161 (±0.4%)

^a^ As determined from the amino acid sequence via ExPASy ProtParam tool [28]. ^b^ According to SEC-MALS.

**Table 2:** PIPE PCR parameters and primers for amplification of four fragments that then self-assemble into the vector pVitro1-hygro carrying M0418 IgE, IgG_1_ or IgG_4_.

PIPE Fragment	Template	Fwd Primer (5′ → 3′)	Rev Primer (5′ → 3′)	Approx. Size (bp)	Extension Time (s)
1a (IgE)	M0418H	M0418H_f GCGGCCGCCACAGGCGCGCACTCCCAGGTGCAGCTGGTGCAGAGC	M0418H_E_r ACGGATGGGCTCTGTGTGCTAGCGCTGCTCACGGTCACGGTGGTG	381	5
	(in pEX_A128)				
1b (IgG_1_)	M0418H	M0418H_f GCGGCCGCCACAGGCGCGCACTCCCAGGTGCAGCTGGTGCAGAGC	M0418H_G1_r GACCGATGGGCCCTTGGTGCTAGCGCTGCTCACGGTCACGGTGGTG	381	5
	(in pEX_A128)				
1c (IgG_4_)	M0418H	M0418H_f GCGGCCGCCACAGGCGCGCACTCCCAGGTGCAGCTGGTGCAGAGC	M0418H_G4_r GGATGGGCCCTTGGTGCTAGCGCTGCTCACGGTCACGGT	381	5
	(in pEX_A128)				
2a (IgE)	ε	E_f GCTAGCACACAGAGCCCATCCGTCTTCCCCTTGACCCGCTGCTGCA	pVitro1L_r ACCGCGGCTAGCTGGAACCCAGAGCAGCAGAAACCCAATGAGTTG	4076	56
	(in pVitro1-hygro-mcs)				
2b (IgG_1_)	γ_1_	G1_f GCTAGCACCAAGGGCCCATCGGTCTTCCCCCTGGCACCCT	pVitro1L_r ACCGCGGCTAGCTGGAACCCAGAGCAGCAGAAACCCAATGAGTTG	3782	52
	(in pVitro1-hygro-mcs)				
2c (IgG_4_)	γ_4_	G4_f GCTAGCACCAAGGGCCCATCCGTCTTCCCCCTGGC	pVitro1L_r ACCGCGGCTAGCTGGAACCCAGAGCAGCAGAAACCCAATGAGTTG	3773	52
	(in pVitro1-hygro-mcs)				
3 (all isotypes)	M0418L	M0418L_f GCTCTGGGTTCCAGCTAGCCGCGGTAGCTACGAGCTGACCCAGCCT	M0418L_K_r CGCCGCCACCGTACGCTTGCCCAGCACGGTCAGCTTGGTG	333	5
	(in pEX_A128)				
4 (all isotypes)	κ	K_f CGTACGGTGGCGGCGCCATCTGTCTTCATCTTCCCGCCATCTG	pVitro1H_r GGAGTGCGCGCCTGTGGCGGCCGCCACCAAGAAGAGGATC	4123	56
	(in pVitro1-hygro-mcs)				

## Supplementary Materials

Supplementary materials can be found at https://www.mdpi.com/1422-0067/21/16/5693/s1.

## Abbreviations

AIT	Allergen immunotherapy
Bet v 1	Major birch pollen allergen from *Betula verrucosa*
BLG	β-lactoglobulin
CD	circular dichroism
CD23	Cluster of differentiation 23, the low-affinity Fc epsilon receptor II
D. I. T.	Digital integration time
DMSO	Dimethyl sulphoxide
ELISA	Enzyme-linked immunosorbent assay
FAP	Facilitated antigen presentation
FBS	Fetal bovine serum
FcεR	Fc epsilon receptor
FSC	Forward scatter
GMFI	Geometric mean fluorescence intensity
HBSS	Hank’s Balanced Salt Solution
Ig	Immunoglobulin
ISAC	Immuno-Solid phase Allergy Chip
kDa	Kilo Dalton
IL	Interleukin
LAD	Laboratory of allergic diseases
PCR	Polymerase chain reaction
PBS	Phosphate-buffered saline
PIPE	Polymerase incomplete primer extension
RBL	Rat basophilic leukaemia
rhSCF	Recombinant human stem cell factor
SDS-PAGE	Sodium dodecyl sulfate-polyacrylamide gel electrophoresis
SEC-MALS	Size exclusion chromatography with multi-angle light scattering
SSC	Side scatter
TBS	Tris-buffered saline

## Appendix A

**Table A1:** Comparison of Key Parameters of Bet v 1 Inhibition ELISAs.

Blocking	Immobilised Bet v 1	Amount of Blocking Protein	Serum	Inhibition
Protein	(pmol/well)	(pmol/well)	Dilution	(%)
M0418 IgG	10	1–100	1:2	20–50 ^a^
H3-1 ScFv [42]	5.8	71.4	1:5	45 ^b^
5B4 IgG_1_ [43]	14.7	0.007–130 ^c^	1:16 ^d^	20–50 ^c^

^a^ Effective blocking with 100 pmol/well; IgG_1_ performed better than IgG_4_. ^b^ Mean of 30 sera; inhibition ranging from 0–62.9%. ^c^ Estimated from graph; effective blocking with ≥0.5 pmol/well. ^d^ Serum pool of 16 donors, diluted and co-incubated with blocking protein.

**Table A2:** Nucleotide sequences of M0418 IgE, IgG_1_ and IgG_4_ light and heavy chains.

**Light chain sequence:**	*ATGTTGCCATCACAACTCATTGGGTTTCTGCTGCTCTGGGTTCCAGCTAGCCGCGGT* **AGCTACGAGCTGACCCAGCCTCCTAGCGCCAGCGGCACCCCTGGCCAGAGAGTGACCATCAGCTGCAGCGGCAGCAGCAGCAACATCGGCGGCAACACCGTGAACTGGTACCAGCAGGTGCCCGGCACCGCCCCTAGACTGCTGATCTACAAGAACAACCAGAGACCCAGCGGCGTGCCCGACAGATTCAGCGGCAGCAAGAGCGGCACCAGCGCCAGCCTGGCCATCAGCGGCCTGAGAAGCGAGGACGAGGCCGACTACTACTGCGAGGCCTGGGACGGCGGCCTGAGAGGCGGCGTGTTCGGCGGCGGCACCAAGCTGACCGTGCTGGGC** AAGCGTACGGTGGCGGCGCCATCTGTCTTCATCTTCCCGCCATCTGATGAGCAGTTGAAATCTGGAACTGCCTCTGTTGTGTGCCTGCTGAATAACTTCTATCCCAGAGAGGCCAAAGTACAGTGGAAGGTGGATAACGCCCTCCAATCGGGTAACTCCCAGGAGAGTGTCACAGAGCAGGACAGCAAGGACAGCACCTACAGCCTCAGCAGCACCCTGACGCTGAGCAAAGCAGACTACGAGAAACACAAAGTCTACGCCTGCGAAGTCACCCATCAGGGCCTGAGCTCGCCCGTCACAAAGAGCTTCAACAGGGGAGAGTGT ***TAG***
*Italic: human leader,*	
**bold: M0418 V_L_ sequence**,	
underlined: hu κ sequence,	
***bold italic: stop codon***	
**IgE heavy chain sequence:**	*ATGGACTGGACCTGGAGGATCCTCTTCTTGGTGGCGGCCGCCACAGGCGCGCACTCC* **CAGGTGCAGCTGGTGCAGAGCGGCGCCGAGGTGAAGAAGCCCGGCGAGAGCCTGAAGATCAGCTGCAAGGGCAGCGAGTACAGCTTCCCCAACTACTGGATCGCCTGGGTGAGACAGATGCCCGGCAAGGGCCTGGAGTGGATGGGCATGATCTACCCCGGCGACAGCGACACCAGATACAGCCCCAGCTTCCAGGGCCAGGTGAACATCAGCGCCGACAAGAGCAGCAGAACCGCCTTCCTGGAGTGGAGCAGCCTGAAGGCCAGCGACAGCGCCACCTACTTCTGCGCCAGACTGGGCGGCCAGCTGTGGAACAGCTACTACTACTACTACTACATGGACGTGTGGGGCAAGGGCACCACCGTGACCGTGAGCAGC** GCTAGCACACAGAGCCCATCCGTCTTCCCCTTGACCCGCTGCTGCAAAAACATTCCCTCCAATGCCACCTCCGTGACTCTGGGCTGCCTGGCCACGGGCTACTTCCCGGAGCCGGTGATGGTGACCTGGGACACAGGCTCCCTCAACGGGACAACTATGACCTTACCAGCCACCACCCTCACGCTCTCTGGTCACTATGCCACCATCAGCTTGCTGACCGTCTCGGGTGCGTGGGCCAAGCAGATGTTCACCTGCCGTGTGGCACACACTCCATCGTCCACAGACTGGGTCGACAACAAAACCTTCAGCGTCTGCTCCAGGGACTTCACCCCGCCCACCGTGAAGATCTTACAGTCGTCCTGCGACGGCGGCGGGCACTTCCCCCCGACCATCCAGCTCCTGTGCCTCGTCTCTGGGTACACCCCAGGGACTATCAACATCACCTGGCTGGAGGACGGGCAGGTCATGGACGTGGACTTGTCCACCGCCTCTACCACGCAGGAGGGTGAGCTGGCCTCCACACAAAGCGAGCTCACCCTCAGCCAGAAGCACTGGCTGTCAGACCGCACCTACACCTGCCAGGTCACCTATCAAGGTCACACCTTTGAGGACAGCACCAAGAAGTGTGCAGATTCCAACCCGAGAGGGGTGAGCGCCTACCTAAGCCGGCCCAGCCCGTTCGACCTGTTCATCCGCAAGTCGCCCACGATCACCTGTCTGGTGGTGGACCTGGCACCCAGCAAGGGGACCGTGAACCTGACCTGGTCCCGGGCCAGTGGGAAGCCTGTGAACCACTCCACCAGAAAGGAGGAGAAGCAGCGCAATGGCACGTTAACCGTCACGTCCACCCTGCCGGTGGGCACCCGAGACTGGATCGAGGGGGAGACCTACCAGTGCAGGGTGACCCACCCCCACCTGCCCAGGGCCCTCATGCGGTCCACGACCAAGACCAGCGGCCCGCGTGCTGCCCCGGAAGTCTATGCGTTTGCGACGCCGGAGTGGCCGGGGAGCCGGGACAAGCGCACCCTCGCCTGCCTGATCCAGAACTTCATGCCTGAGGACATCTCGGTGCAGTGGCTGCACAACGAGGTGCAGCTCCCGGACGCCCGGCACAGCACGACGCAGCCCCGCAAGACCAAGGGCTCCGGCTTCTTCGTCTTCAGCCGCCTGGAGGTGACCAGGGCCGAATGGGAGCAGAAAGATGAGTTCATCTGCCGTGCAGTCCATGAGGCAGCGAGCCCCTCACAGACCGTCCAGCGAGCGGTGTCTGTAAATCCCGGTAAA ***TGA***
*Italic: human leader,*	
**bold: M0418 V_H_ sequence**,	
underlined: hu ε sequence,	
***bold italic: stop codon***	
**IgG_1_ heavy chain sequence:**	*ATGGACTGGACCTGGAGGATCCTCTTCTTGGTGGCGGCCGCCACAGGCGCGCACTCC* **CAGGTGCAGCTGGTGCAGAGCGGCGCCGAGGTGAAGAAGCCCGGCGAGAGCCTGAAGATCAGCTGCAAGGGCAGCGAGTACAGCTTCCCCAACTACTGGATCGCCTGGGTGAGACAGATGCCCGGCAAGGGCCTGGAGTGGATGGGCATGATCTACCCCGGCGACAGCGACACCAGATACAGCCCCAGCTTCCAGGGCCAGGTGAACATCAGCGCCGACAAGAGCAGCAGAACCGCCTTCCTGGAGTGGAGCAGCCTGAAGGCCAGCGACAGCGCCACCTACTTCTGCGCCAGACTGGGCGGCCAGCTGTGGAACAGCTACTACTACTACTACTACATGGACGTGTGGGGCAAGGGCACCACCGTGACCGTGAGCAGC** GCTAGCACCAAGGGCCCATCGGTCTTCCCCCTGGCACCCTCCTCCAAGAGCACCTCTGGGGGCACAGCGGCCCTGGGCTGCCTGGTCAAGGACTACTTCCCCGAACCGGTGACGGTGTCGTGGAACTCAGGCGCCCTGACCAGCGGCGTGCACACCTTCCCGGCTGTCCTACAGTCCTCAGGACTCTACTCCCTCAGCAGCGTGGTGACCGTGCCCTCCAGCAGCTTGGGCACCCAGACCTACATCTGCAACGTGAATCACAAGCCCAGCAACACCAAGGTGGACAAGAAAGTTGAGCCCAAATCTTGTGACAAAACTCACACATGCCCACCGTGCCCAGCACCTGAACTCCTGGGGGGACCGTCAGTCTTCCTCTTCCCCCCAAAACCCAAGGACACCCTCATGATCTCCCGGACCCCTGAGGTCACATGCGTGGTGGTGGACGTGAGCCACGAAGACCCTGAGGTCAAGTTCAACTGGTACGTGGACGGCGTGGAGGTGCATAATGCCAAGACAAAGCCGCGGGAGGAGCAGTACAACAGCACGTACCGTGTGGTCAGCGTCCTCACCGTCCTGCACCAGGACTGGCTGAATGGCAAGGAGTACAAGTGCAAGGTCTCCAACAAAGCCCTCCCAGCCCCCATCGAGAAAACCATCTCCAAAGCCAAAGGGCAGCCCCGAGAACCACAGGTGTACACCCTGCCCCCATCCCGGGATGAGCTGACCAAGAACCAGGTCAGCCTGACCTGCCTGGTCAAAGGCTTCTATCCCAGCGACATCGCCGTGGAGTGGGAGAGCAATGGGCAGCCGGAGAACAACTACAAGACCACGCCTCCCGTGCTGGACTCCGACGGCTCCTTCTTCCTCTACAGCAAGCTCACCGTGGACAAGAGCAGGTGGCAGCAGGGGAACGTCTTCTCATGCTCCGTGATGCATGAGGCTCTGCACAACCACTACACGCAGAAGAGCCTCTCCCTGTCTCCGGGTAAA ***TGA***
*Italic: human leader,*	
**bold: M0418 V_H_ sequence**,	
underlined: hu γ_1_ sequence,	
***bold italic: stop codon***	
**IgG_4_ heavy chain sequence**	*ATGGACTGGACCTGGAGGATCCTCTTCTTGGTGGCGGCCGCCACAGGCGCGCACTCC* **CAGGTGCAGCTGGTGCAGAGCGGCGCCGAGGTGAAGAAGCCCGGCGAGAGCCTGAAGATCAGCTGCAAGGGCAGCGAGTACAGCTTCCCCAACTACTGGATCGCCTGGGTGAGACAGATGCCCGGCAAGGGCCTGGAGTGGATGGGCATGATCTACCCCGGCGACAGCGACACCAGATACAGCCCCAGCTTCCAGGGCCAGGTGAACATCAGCGCCGACAAGAGCAGCAGAACCGCCTTCCTGGAGTGGAGCAGCCTGAAGGCCAGCGACAGCGCCACCTACTTCTGCGCCAGACTGGGCGGCCAGCTGTGGAACAGCTACTACTACTACTACTACATGGACGTGTGGGGCAAGGGCACCACCGTGACCGTGAGCAGC** GCTAGCACCAAGGGCCCATCCGTCTTCCCCCTGGCGCCCTGCTCCAGGAGCACCTCCGAGAGCACAGCCGCCCTGGGCTGCCTGGTCAAGGACTACTTCCCCGAACCGGTGACGGTGTCGTGGAACTCAGGCGCCCTGACCAGCGGCGTGCACACCTTCCCGGCTGTCCTACAGTCCTCAGGACTCTACTCCCTCAGCAGCGTGGTGACCGTGCCCTCCAGCAGCTTGGGCACGAAGACCTACACCTGCAACGTAGATCACAAGCCCAGCAACACCAAGGTGGACAAGAGAGTTGAGTCCAAATATGGTCCCCCATGCCCATCATGCCCAGCACCTGAGTTCCTGGGGGGACCATCAGTCTTCCTGTTCCCCCCAAAACCCAAGGACACTCTCATGATCTCCCGGACCCCTGAGGTCACGTGCGTGGTGGTGGACGTGAGCCAGGAAGACCCCGAGGTCCAGTTCAACTGGTACGTGGATGGCGTGGAGGTGCATAATGCCAAGACAAAGCCGCGGGAGGAGCAGTTCAACAGCACGTACCGTGTGGTCAGCGTCCTCACCGTCCTGCACCAGGACTGGCTGAACGGCAAGGAGTACAAGTGCAAGGTCTCCAACAAAGGCCTCCCGTCCTCCATCGAGAAAACCATCTCCAAAGCCAAAGGGCAGCCCCGAGAGCCACAGGTGTACACCCTGCCCCCATCCCAGGAGGAGATGACCAAGAACCAGGTCAGCCTGACCTGCCTGGTCAAAGGCTTCTACCCCAGCGACATCGCCGTGGAGTGGGAGAGCAATGGGCAGCCGGAGAACAACTACAAGACCACGCCTCCCGTGCTGGACTCCGACGGCTCCTTCTTCCTCTACAGCAGGCTAACCGTGGACAAGAGCAGGTGGCAGGAGGGGAATGTCTTCTCATGCTCCGTGATGCATGAGGCTCTGCACAACCACTACACACAGAAGAGCCTCTCCCTGTCTCTGGGTAAA ***TGA***
*Italic: human leader,*	
**bold: M0418 V_H_ sequence**,	
underlined: hu γ_4_ sequence,	
***bold italic: stop codon***	
