# Supplementary material for: Filling the Antibody Pipeline in Allergy: PIPE Cloning of IgE, IgG1 and IgG4 against the Major Birch Pollen Allergen Bet v 1
Source: Int J Mol Sci. 2020 Aug 8;21(16):5693. doi: 10.3390/ijms21165693 (PMC7460837; doi:10.3390/ijms21165693)
Supplement: Supplementary file 1 [file ijms-21-05693-s001.pdf]

International Journal of *Molecular Sciences*

MDPI

Supplementary Material – Uncropped Gel Images

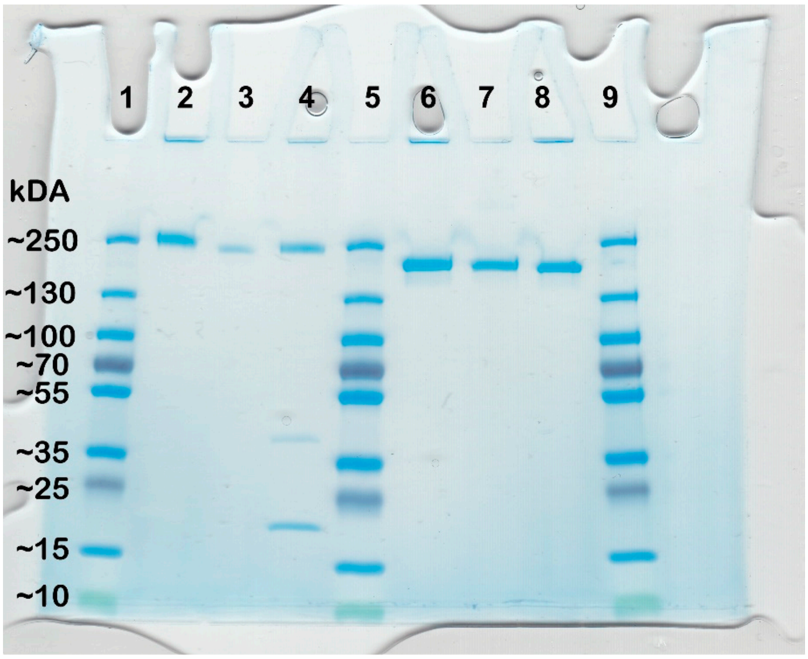

Figure S1. Uncropped gel image of SDS–PAGE, non–reducing conditions. 1, Marker; 2, M0418 IgE; 3, Isotype IgE; 4, unrelated IgE; 5, Marker; 6, M04128 IgG_1; 7, isotype IgG_1; 8, unrelated IgG_1; 9, Marker.

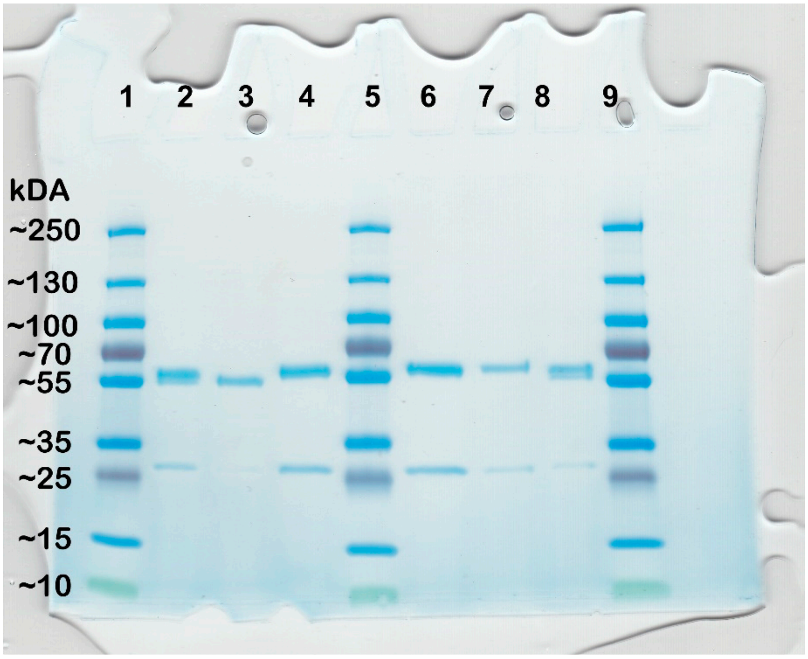

Figure S2. Uncropped gel image of SDS–PAGE, reducing conditions. 1, Marker; 2, M0418 IgG_1; 3, Isotype IgG_1; 4, unrelated IgG_1; 5, Marker; 6, M04128 IgG_4; 7, isotype IgG_4; 8, unrelated IgG_4; 9, Marker

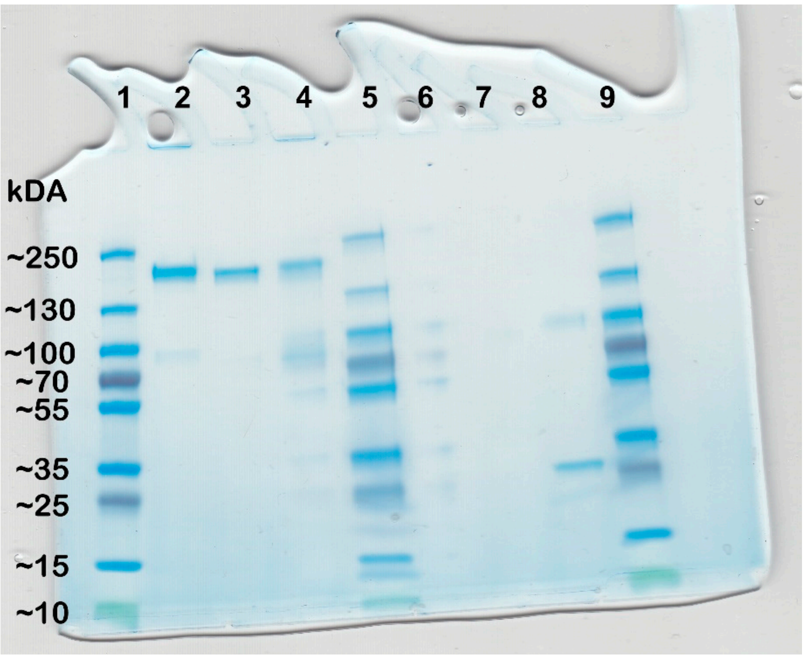

Figure S3. Uncropped gel image of SDS–PAGE, non–reducing conditions (left); 1, Marker; 2, M0418 IgG_4; 3, Isotype IgG_4; 4, unrelated IgG_4; 5–9: irrelevant, was repeated.

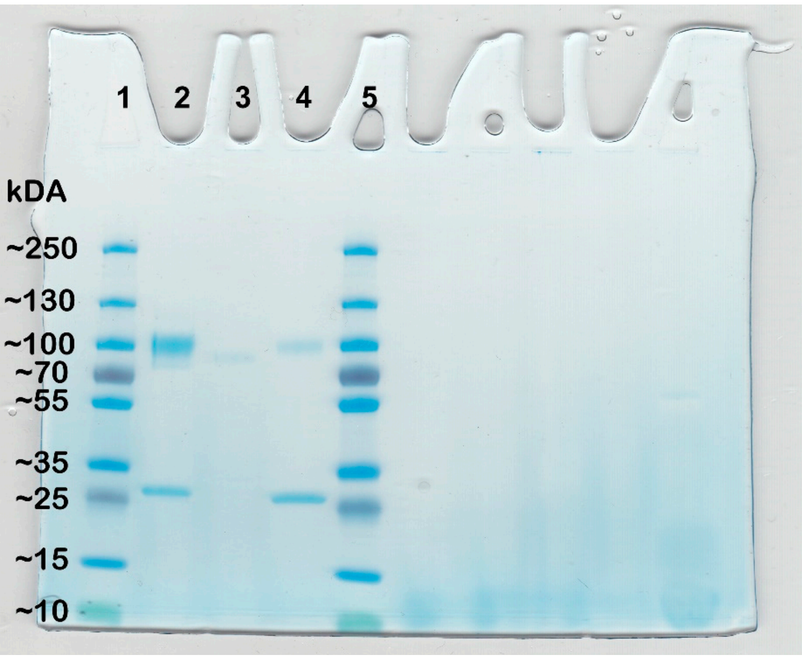

Figure S4. Uncropped gel image of SDS–PAGE, reducing conditions. 1, Marker; 2, M0418 IgE; 3, Isotype IgE; 4, unrelated IgE; 5, Marker.

Supplementary Material – FcεRI and CD23 Expression of U937 and RPMI–8866 Cells

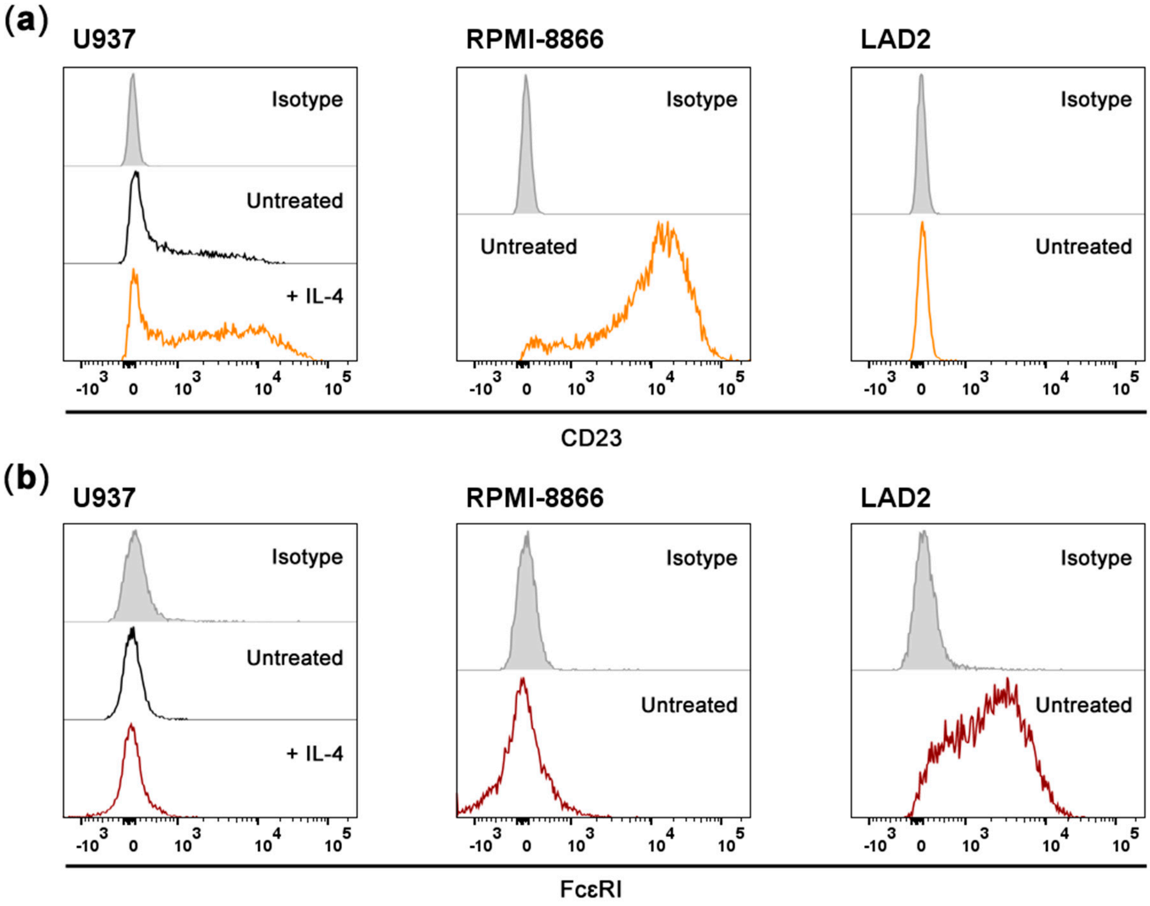

Figure S5. IgE receptor expression of model cell lines U937 and RPMI–8866. (a) U937 monocytes express a moderate level of the low–affinity receptor CD23, which is enhanced by priming with IL–4. RPMI–8866 cells express high levels of CD23, whereas the control mast cell line LAD2 is CD23–negative. (b) U937 monocytes and RPMI–8866 cells are high–affinity receptor FcεRI–negative, as compared to the control mast cell line LAD2.
